# Combination of photodynamic therapy (PDT) and anti-tumor immunity in cancer therapy

**DOI:** 10.1007/s40005-017-0377-x

**Published:** 2018-11-01

**Authors:** Hee Sook Hwang, Heejun Shin, Jieun Han, Kun Na

**Affiliations:** Department of Biotechnology, The Catholic University of Korea, 43 Jibong-ro, Wonni-gu, Bucheno-si, Gyeonggido 14662 South Korea

**Keywords:** Photodynamic therapy, Reactive oxygen species, Cancer, Antitumor immune response

## Abstract

Photodynamic therapy (PDT) is performed using a photosensitizer and light of specific wavelength in the presence of oxygen to generate singlet oxygen and reactive oxygen species(ROS) in the cancer cells. The accumulated photosensitizers in target sites induce ROS generation upon light activation, then the generated cytotoxic reactive oxygen species lead to tumor cell death via apoptosis or necrosis, and damages the target sites which results tumor destruction. As a consequence, the PDT-mediated cell death is associated with anti-tumor immune response. In this paper, the effects of PDT and immune response on tumors are reviewed. Activation of an immune response regarding the innate and adaptive immune response, interaction with immune cells and tumor cells that associated with antitumor efficacy of PDT are also discussed.

## Introduction

**Fig. 1:**
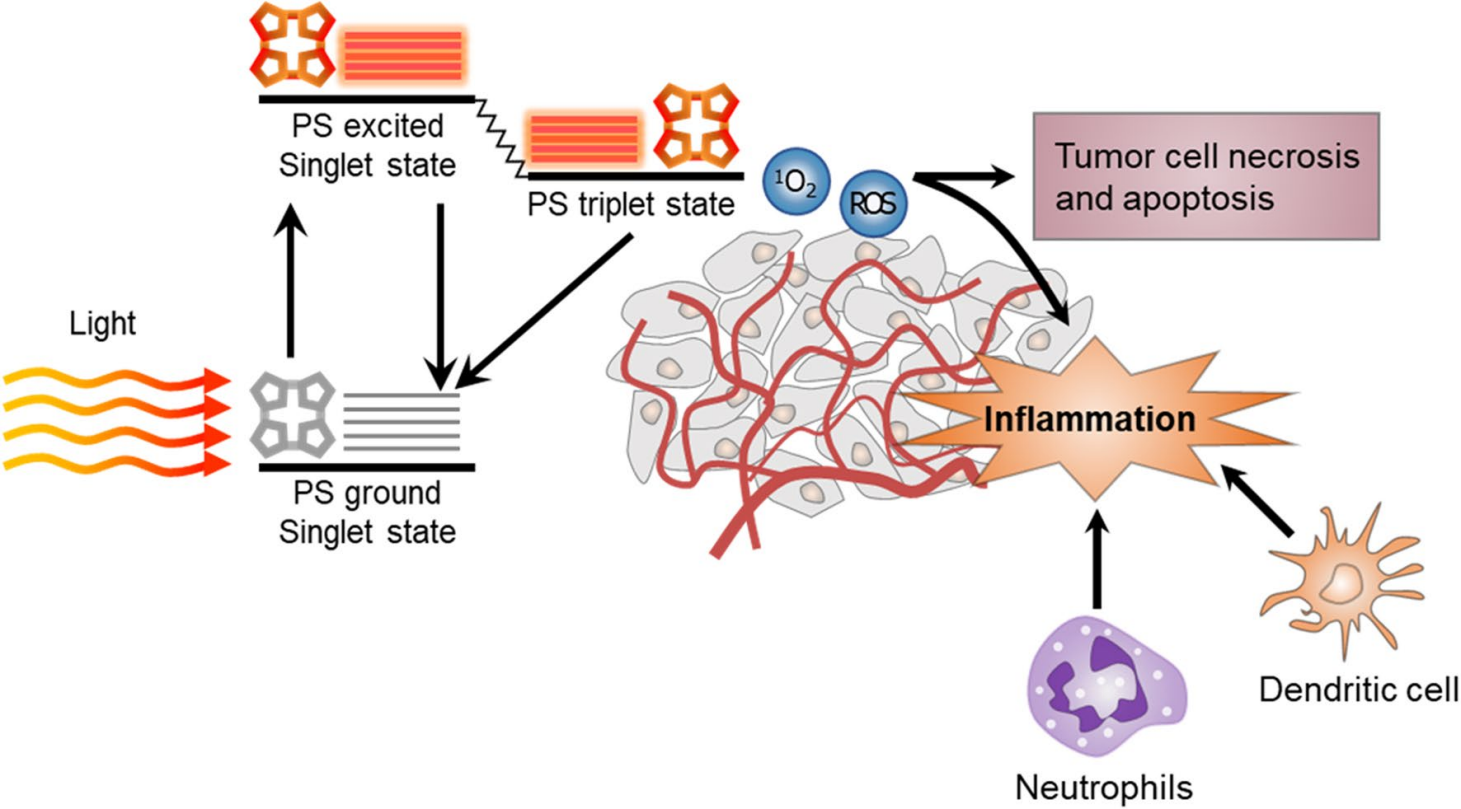
On the mechanism of the anti-tumor response induced by photodynamic therapy. (Modified with permission from *Nat Rev Cancer* Copyright 2006)

Photodynamic therapy (PDT) is a minimally invasive treatment that has been applied for clinical use in various diseases such as intraepithelial neoplasias, glioblastoma, and cancer therapy (Yang et al. 2016; Sanabria et al. 2013; Brown et al. 2004). The clinical potential of PDT has been recognized more than 25 years, and PDT with porfimer sodium was first approved in 1995 to treat lung, gastric, cervical, and bladder cancer, aminolevulinic acid was approved in 1999 to treat actinic keratosis, and temoporfin was approved in 2001 for palliative head and neck cancer therapy (Brown et al. 2004). In PDT, a nontoxic photosensitizer absorbs light and excites along with electron transfer which involves series of photochemical reactions and produces conversion of reactive singlet oxygen to highly reactive oxygen species (ROS) (Fig. 1) (Castano et al. 2006).

PDT provides several advantages over the conventional cancer therapy which includes less invasive than surgery, precise tumor targeting, minimal systemic toxicity, and availability of repeated treatments (Svensson et al. 2007; Yang et al. 2016; Sanabria et al. 2013). However, PDT still have drawbacks because of limitations of light penetration into deep tumor tissues, development of skin photosensitivity after treatment, and difficulty to treat metastatic cancers (Agostinis et al. 2011). Nonetheless, PDT has been developed as a powerful tool to induce antitumor immune responses. The influence of PDT on the immune response is involved in acute inflammatory response, leukocyte infiltration of the tumor, and production of proinflammatory cytokines (Yang et al. 2016).

## PDT-mediated tumor destruction

**Fig. 2:**
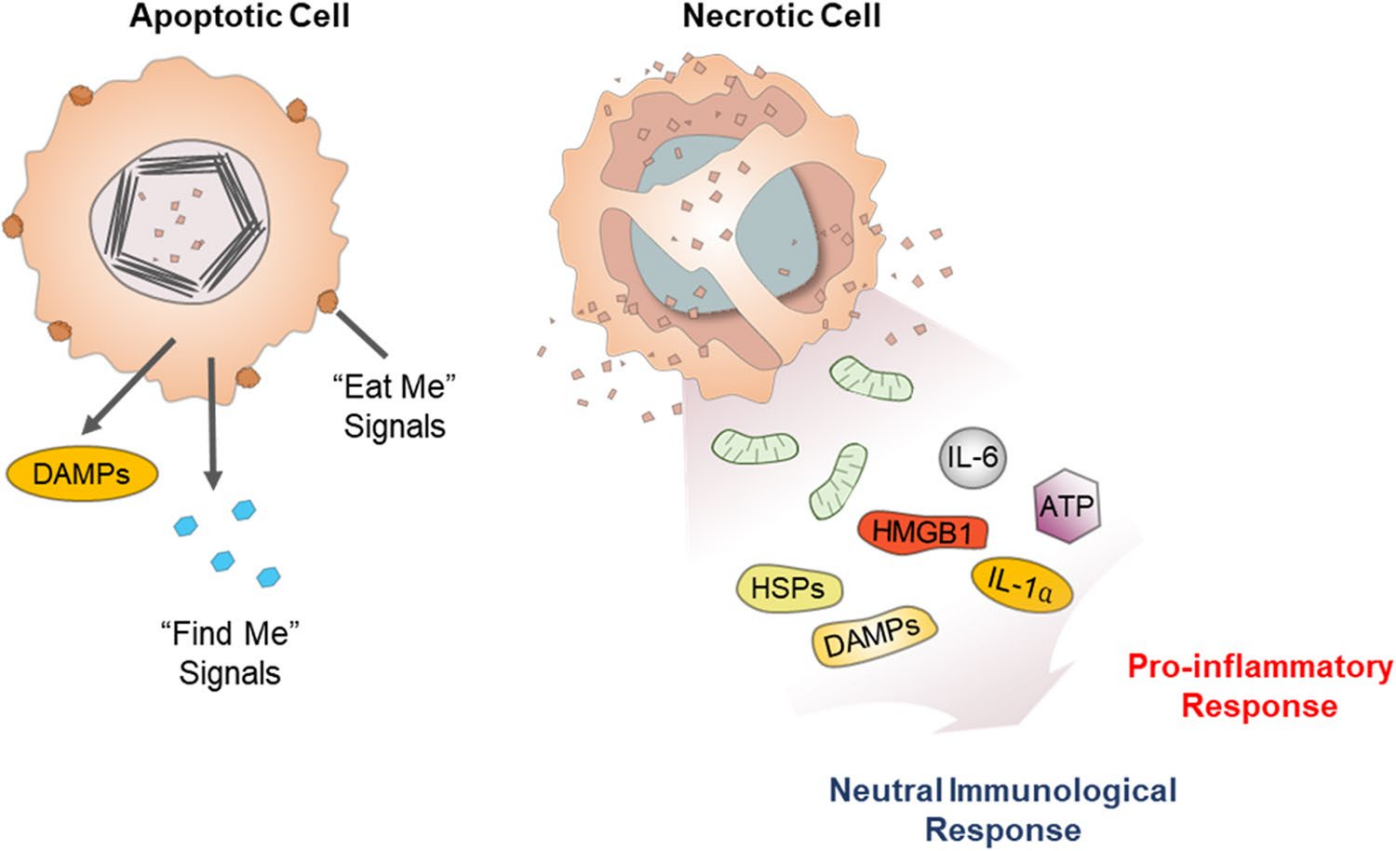
Two major cell death morphotypes and their immunological profiles. (Modified with permission from *Apoptosis* Copyright 2010)

Antitumor effects of PDT on tumors are involving three main mechanisms to destruct tumors: three mechanisms include tumor cell death via ROS, tumor-associated vasculature damage, and initiation of immune response against tumor cells (Fig. 2) (Dolmans et al. 2002).

### Direct tumor cell killing due to cytotoxic ROS

PDT-treated cells are subjected to cell death either by apoptosis or necrosis. Necrosis is unprogrammed process that also called accidental cell death. Necrotic cells swell and disrupt the plasma membrane that results the release of intracellular components including proinflammatory molecules that leads to inflammatory reaction. (Robertson et al. 2009). Whereas, apoptosis is a controlled and energy-consuming process that results suicide cell death. It is another type of dominant form of cell death that resulted by PDT. PDT-induced apoptotic cells activate endonuclease that degrades DNA into oligonucleosomal fragments and leads to caspases activation (Robertson et al. 2009). It shows two different apoptosis mechanisms such as intrinsic/mitochondria-mediated apoptosis and extrinsic/death receptor-mediated apoptosis.

#### Intrinsic/mitochondria-mediated apoptosis

The mitochondrial apoptosis pathway involves release of two proteins; cytochrome c and apoptosis-inducing factor from the intermembrane space into the cytosol (Lam et al. 2001). The generation of ROS in mitochondria via PDT initializes mitochondrial inner membrane permeabilization and activates mitochondrial apoptotic death. Mitochondrial membrane permeabilization is controlled by Bcl-2 family members (Garg et al. 2010; Nowis et al. 2005).

#### Extrinsic/death receptor-mediated apoptosis

Death receptor-mediated apoptosis occurs when photosensitizers target the cell membrane, and this pathway is triggered by cell surface death receptors which belong to the tumor necrosis factor (TNF) receptor (Nowis et al. 2005). PDT-induced death receptor-mediated apoptosis is involved with cytochrome c release and caspase activation in cells (Nowis et al. 2005).

### Tumor vascular damage caused by generated ROS

Laser irradiation of the tumor areas by specific light wavelength generates highly cytotoxic ROS which damages tumor cells and vessels. More in details, ROS generates irreversible damages in endothelial cells and the vascular basement membrane that affects vasoactive molecules release, vascular permeability, and vessel constriction. The collapse of vasculature and tissue hemorrhages lead to tumor destruction (Krammer 2000). PDT-mediated damage to the vasculature is initiation of inflammatory response in tumor. Since tumor growth is related to the function of vasculature due to the oxygen and nutrients supply, microvasculature destruction and prevention of the blood vessel formation damage tumor blood vessels, result blood vessel occlusion and hemorrhages, and kill tumor cells (Korbelik 1996; Bhuvaneswari et al. 2009). It has been known that PDT damages tumor-associated vasculature and many studies reported that there is influence of PDT on the tumor vasculature and its impact on tumor cells. Dolmans group proved that PS-light intervals mainly target tumor vasculature using a dose of MV6401 photosensitizer. Short intervals between MV6401 administration and light delayed on orthotopic breast tumor growth, since MV6401 accumulation in the tumor tissue induced vascular shutdown followed by tumor cell death. They suggested that fractionated drug dose improved anti-vascular effects because of the targeting of vasculature and tumors by PDT (Chen et al. 2006).

### Local inflammatory response

The effects of PDT are involved in destruction of tumor and vasculature that induce local inflammatory response. Phototoxic damage of tumor cell membrane acts as the inflammatory mediators which is involved in initiation of the acute inflammatory reaction (Agostinis et al. 2011; Korbelik 1996). As a consequence, the damaged areas locally produce proinflammatory cytokines and chemokines, and play important roles in development of innate and adaptive immune response which will be covered in following sections with details. PDT inflammation also involves leukocyte infiltration into the target sites which includes neutrophils, mast cells, monocytes and macrophages (Gollnick et al. 2003). PDT-mediated cell death causes tumor antigens release along with increased supply of cell death-associated molecular patterns (CDAMs) or damage associated molecular patterns (DAMPs) that have immunostimulatory properties. These CDAMs and DAMPs from dying tumors develop acute inflammatory response. Herein, the immune response caused by the PDT was focused.

## PDT and immune response

### Local inflammatory response

PDT generates significant effect on the immune system (Castano et al. 2006; Sanabria et al. 2013). PDT induced cell death generates a strong and acute inflammatory reaction. The local inflammatory response leads to neutrophil and inflammatory cell accumulation at the treated sites to attack tumor cells (Mroz et al. 2011). Initiation of light treatments result rapid recruitment of neutrophils (Korbelik 1996). This immune system also involves the expression of transcription factors including AP-1 and NF-κB which lead to expression of cytokines, adhesion molecules, leukocytes, and interleukins in later (Nowis et al. 2005). The inflammatory response slowly develops to adaptive immunity followed by systemic immunity induction.

### Systemic inflammation

Maturation and activation of dendritic cells (DC) increase DC activation and enhance PDT generated anti-tumor immunity (Brackett and Gollnick 2011; Sanabria et al. 2013). The maturated DC then migrates to lymph nodes where tumor-associated antigens (TAA) peptides are present with major histocompatibility complex (MHC) class I and II to CD8+ and CD4+ T cells, respectively. (Nowis et al. 2005). In addition, tumor cell lysates generate interleukin (IL) 1α/β, IL-6, IL-8, and TNF-α secretion from immune cells, and especially IL-1 and IL-6 play an important role in inflammatory regulation process (Nowis et al. 2005; Agostinis et al. 2011). Since PDT treatment is associated with numerous cytokines generation, there have been many studies that measure cytokines in serum after PDT and demonstrate increased levels of proinflammtory cytokines (Table 1).

**Table 1:** PDT generated proinflammatory cytokines

Cytokines	Secreted immune cells	Immunomodulatory function	Ref.
IL-1 α/β	Macrophages, DC, stromal cells, B cells	Highly inflammatory cytokine which upregulate host defense and function as an immunoadjuvant	Dinarello (1997)
IL-6	Macrophages, stromal cells, T cells, B cells	Activator of immune system that involved in transition from innate to adaptive immunity	Scheller et al. (2011), Berghe et al. (2006)
IL-8	Monocytes Bone marrow	Chemoattractant cytokine that has target specificity for neutrophil and activate neutrophils in inflammatory areas	Bickel (1993)
TNF-α	Macrophages, stromal cells, mast cells, lymphocytes	Promote T cell activation and increase adaptive antitumor immunity	Brackett and Gollnick (2011)

**Fig. 3:**
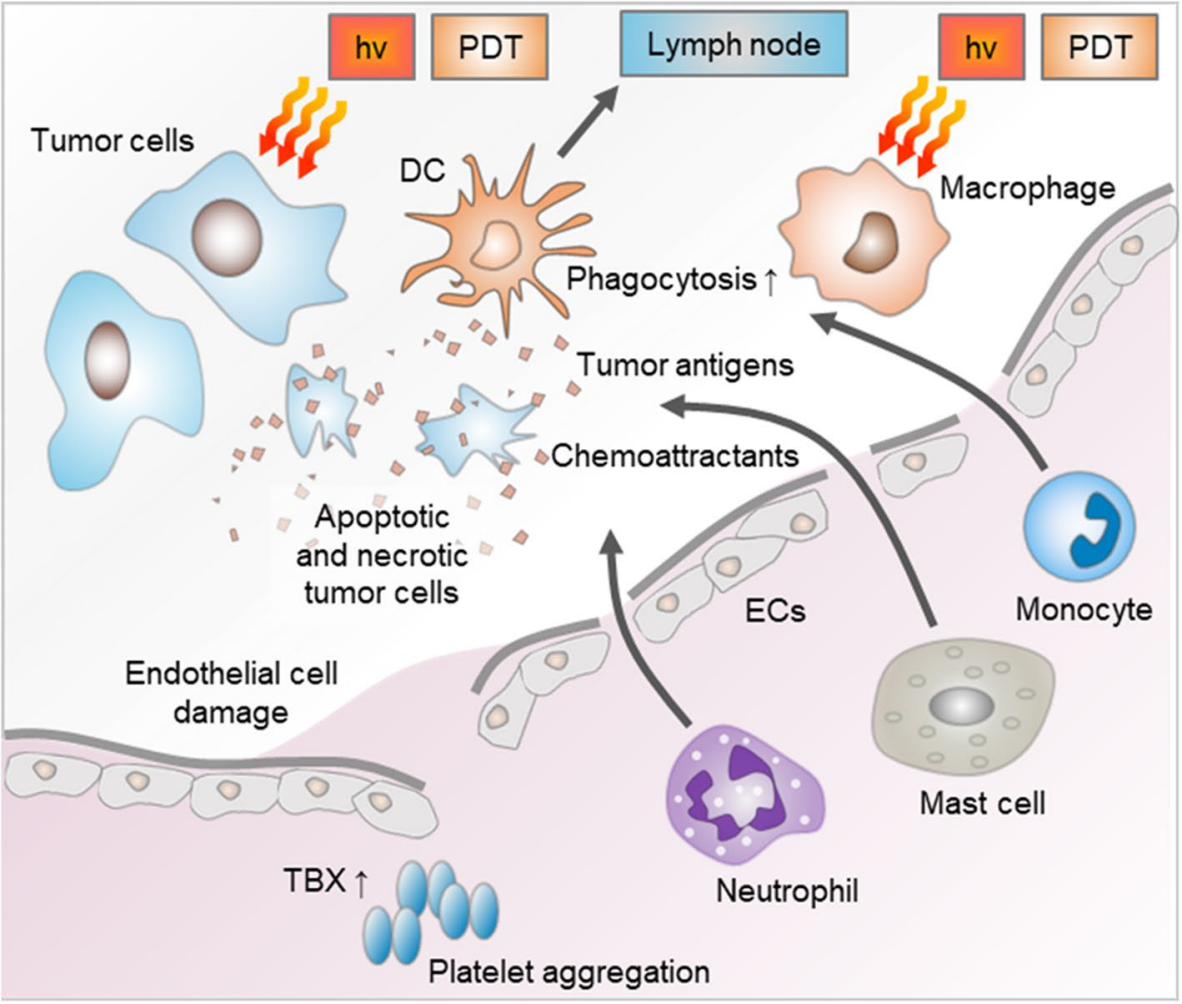
Photodynamic therapy induced inflammatory response. (Modified with permission from *Nat Rev Cancer* Copyright 2006)

PDT-treated dying cells are also involved in expression of heat shock proteins (HSPs) to the cell surface after PDT treatment and stimulates an anti-tumor immune response. HSPs are family of protein chaperons that assist protein folding and unfolding. PDT treated dying cells increase HSP70 expression by cellular stress (Helbig et al. 2011; Garg et al. 2010). Regarding the expression of HSPs, several HSPs are secreted from cells associated after PDT treatment (Sanabria et al. 2013). The most important HPSs are members of HSP 70, which is one of the anti-apoptotic proteins and a major PDT generated danger signal (Helbig et al. 2011; Korbelik 2006; Garg et al. 2010). PDT treatment of solid tumors provoke the upregulation of Hsp70 gene in the host liver and spleen and the levels of Hsp70 expression correlate with the capacity of vaccine cells to stimulate DC maturation and antitumor immune response (Fig. 3) (Merchant and Mladen 2011; Castano et al. 2006).

## Influence of PDT on immune cells

### PDT and innate immune response

The ideal cancer therapy modality is induction of local tumor regression and eradication, as well as a systemic anti-tumor immunity that could effectively eradicate distant metastatic cancer cells without toxic to normal tissue. From this prospective, PDT can be a great alternative since it produces acute inflammation and attracts immune cells to treat distant tumors (Gollnick et al. 2006; Preise et al. 2009; Mroz et al. 2011). PDT elevates oxidative stress significantly at treated tumor sites causing cellular membrane and cytoplasmic structure damages inducing inflammatory response (Mroz et al. 2011). To maintain homeostasis, the host secretes proinflammatory mediators causing activation of complement and accumulation of neutrophils and other inflammatory cells in the treated tumor sites to attack tumor cells (Korbelik 2006).

#### Macrophages

Macrophage is a crucial cell which is responsible for innate immunity. Central functions of macrophage are maintaining homeostasis and host defense through phagocytosis (Korbelik et al. 1997; Wynn et al. 2013). In addition, they can directly cytotoxic to tumor cells as well as engaged in the activation of adaptive immunity through presentation of tumor antigens (TAs) (Mroz et al. 2011). Macrophages can be activated by low sublethal doses of PDT and secret TNF-α, which is a macrophage activating factor. Recent report indicates that macrophages show preferential cytotoxicity towards tumor cells.

#### Neutrophils

Neutrophil is one of the granulocytes that form the innate immune system. Unlike macrophages, its main function is secreting cytokines such as leukotrienes and prostaglandins that cause an inflammatory response rather than phagocytosis (Nathan 2006). Upon PDT application, the local increase of chemokine such as macrophage inflammatory protein-2 and adhesion molecule E-selectin in the PDT treated tumor area results in migration of neutrophil, which promotes CD8+ T cell proliferation (Kousis et al. 2007). The lack of neutrophil disturbs T cell proliferation, which is unable to mount strong anti-tumor CD8+ T cell response after PDT treatment. In this reason, neutrophils play an important role in the anti-tumor immunity upon PDT treatment.

#### Natural killer (NK) cells

To investigate how NK cells are involved in anti-tumor immunity after PDT, Kabingu et al. tested NK cells depletion in EMT/6 tumor-bearing severe combined immunodeficient (SCID) mice (Kabingu et al. 2007). After PDT treatment, they found that NK cells involved in PDT-induced anti-tumor immunity by observing number of lung tumors per mouse were significantly higher than that of NK cells deficient mouse. In addition, PDT was performed by replenished with CD8+ T cells and NK cells deficient SCID mice, and confirmed that SCID mouse exhibited significant increase in lung tumor numbers. This result suggests that NK cells not only play an important role in anti-tumor immunity but also affect activity of CD8+ T cells after PDT treatment, and control distant nontreated metastases.

Dendritic cells (DCs). DCs are the most representative cells in antigen presenting cells (APCs) and play an important role in anti-tumor immunity response development. The tumor micro-environment not only prevents secreting a proinflammatory signal that promotes the DC activation but also provides immunosuppressive mechanism, so that maturation of DC interfered and the function as the APC is lost (Gabrilovich et al. 1996). PDT also promotes DCs maturation and migration to draining lymph nodes by inducing local inflammation (Brackett and Gollnick 2011). This process promotes the activation of CD4 helper T-cell, CD8+ cytotoxic T lymphocyte, and B cells, resulting in an adaptive immune response. A recent report demonstrated that PDT-generated tumor cell lysate induces IL-1α, IL-1β, and IL-6 secretion from DCs suggesting that PDT-induced immune enhancement is due to DCs activation (Ashley et al. 2011).

### PDT and adaptive immunity

Induction of acute inflammation by PDT recruits neutrophils into PDT-treated tumor areas and secretes chemokines and granule proteins to stimulate DCs maturation and activation (Mroz et al. 2011). Activated DCs migrate to lymph nodes, activating T-cells and B-cells, resulting in adaptive immune response (Brackett and Gollnick 2011). Canti and colleagues examined the anti-tumor immune response in both immunosuppressed and normal mice bearing MS-2 fibrosarcomas (Cantl et al. 1994). All mice were cured and survived indefinitely, but there was resistance development of MS-2 rechallenge in normal surviving animals which was cured by PDT, whereas immunosuppressed surviving animals died after tumor rechallenge. This result demonstrates that adaptive immunity is induced by PDT.

Although PDT activates both humoral and cell-mediated adaptive anti-tumor immunity, the importance of the humoral anti-tumor immunity has not been elucidated (Castano et al. (2006; Preise et al. 2009; Brackett and Gollnick 2011). In addition, Gollnick et al. demonstrated that CD4+ T cell depletion had no effect on the ability of PDT on tumors, whereas, the efficacy of PDT was dependent upon CD8+T cells (Kabingu et al. 2007). Korbelik et al. reported that adaptive transfer of splenocytes (mixture of CD4+ and CD8+ T cells with some B cells, NK cells, and monocytes) from normal mice, which cured EMT6 tumors by PDT, to SCID mice resulted in fully restored curative effect of PDT on EMT6 tumors (Mladen Korbelik et al. 1996). They concluded that the depletion of specific T-cell populations from donor splenocytes indicates that curative effect is mostly due to CD8+ T, whereas CD4+ T cell played a supportive role. Table 2 summarizes the immune cells involved in innate and adaptive immunity.

**Table 2:** PDT-mediated immune cells

Innate immunity	Location	Ref.
Macrophages		
• Maintaining homeostasis and host defense through phagocytosis of foreign pathogens and cancer cells • Stimulates response of other immune cells through presentation of tumor antigens	Migrates from blood vessels to tissues	Wynn et al. (2013)
Neutrophils		
• Release toxins that kill or inhibit pathogens and recruits other immune cells to the site of infection • Induce inflammatory response through secreting cytokines and promotes CD8+ T cell proliferation	Migrates from blood vessels to tissues	Nathan (2006)
Natural killer (NK) cells		
• Type of cytotoxic lymphocyte critical to the innate immune response. • Response to infected cells and tumor formation and kills infected cells and tumor cells	Circulates in blood and migrates to tissues	Kabingu et al. (2007)
Dendritic cells (DCs)		
• Present antigens on its surface, thereby triggering adaptive immunity (Antigen presenting cells, APCs)	Present in epithelial tissue, including skin, lung, stomach and intestines. It migrates to lymph nodes upon activation	Mroz et al. (2011)

Adoptive immunity	Location	Ref.
T cell		
CD4+ T cell (Helper T cell)	Thymus	Castano et al. (2006)
• Aid immune responses by releasing signaling molecules known as cytokines (Initiating both cell cytotoxic T cell and B cell responses)		
CD8+ T cell (Cytotoxic T cell)		
• Detect and inducing death to infected somatic or tumor cells		
B cell		
• Produce immunoglobulins, the antigen specific antibodies to eliminate antigens • Antigen presentation	Bone marrow	Castano et al. (2006)

### PDT-generated cancer vaccine

The concept of cancer vaccination mechanism is similar to conventional vaccination which is introduction of attenuated or killed forms of the microbe that body recognizes as foreign and produces protective antibodies against it. Cancer vaccine is produced by exposing tumor cells to lethal radiation doses and then introducing these killed tumor cells to animal with the expectation that host’s immune system will recognize the killed tumor cells and develop immunity (Mroz et al. 2011). Gollnick et al. compared the cancer vaccine potential of PDT-generated cell lysate with lysate generated by UV or ionizing radiation (Gollnick et al. 2002). PDT generated vaccines were tumor specific and induced a cytotoxic T-cell response unlike other methods. The excised lymph nodes from the mice, 4 days post-vaccination with PDT-generated vaccine, B-cells, T-cells, and DCs, were dramatically increased in PDT vaccinated mice compared to controls. This result demonstrates that PDT-derived anticancer vaccine has clinical potentials to become a beneficial adjuvant or primary therapy in treatment of various cancers.

### PDT-mediated immune checkpoint blockade therapy

Recently, immune checkpoint is a field that become a hot topic, and many studies have been actively conducted. Three immune checkpoint agents for melanoma therapy have been approved by the FDA and other drugs will be approved to treat patients with various cancer types including kidney, lung, bladder, and prostate cancer (Sharma and Allison 2015). In 2011, the antibody agent against CTLA-4 (ipilimumab) was approved and 3 years later, other two antibody agents against PD-1 (pembrolizumab and nivolumab) were approved (Min et al. 2017). In this perspective, the immune checkpoint and their blockade, particularly about anti-CTLA-4, anti-PD-1, or anti-PD-L1 will be discussed.

The immune response of antigen-specific T cells is a very complex and elaborate regulatory process. The activation of T cell begins with the recognition that the T cell antigen receptor (TCR) on the surface of T cell binds to the major histocompatibility complex Class (MHC) II molecules in antigen presenting cell (APC) (Topalian et al. 2016). However, for effective activation of T cells, costimulatory signals are required at the same time as recognition of the antigen. This is accomplished by binding the B7 molecules (CD80 and CD86) expressed in APC simultaneously with CD28, ligand on the surface of the T cell, thereby, activating the secretion of cytokines (Topalian et al. 2016). The recognition of the antigen through the binding of TCR-MHC/epitope does not result in the activation of the T cell without costimulatory signaling. However, since the activated T cell is programmed to be deactivated after a predetermined time, the co-inhibition signal is activated which allows the side effects to be avoided due to excessive immune response. Among the various kinds of these co-inhibitory signals, there are typically cytotoxic T lymphocyte antigen (CTLA)-4 and programmed death-ligand 1 (PD-L1) of T cells, and the corresponding ligands are involved in CD86 and PD-L1 on APC (Dong et al. 1999). In addition, when CTLA-4 binds to B7 molecule ligand, it deactivates the naïve or memory T cells, and PD-1 binds to PD-L to regulate T cell function in peripheral tissues (Demaria et al. 2005). The immune system controls the overall T cell activity through regulation of these co-stimulatory and co-inhibitory signals, which is called an immune checkpoint.

The normal immune system detects tumor-specific antigens expressed by changes such as mutations in tumor microenvironment, and affords to remove them (Teng et al. 2015). In contrast, tumor cells evade immune function by altering the tumor microenvironment to avoid immune attacks, immune tolerance, or immuno-editing such as T cell immune evasion. As one of these immune avoidance strategies, tumor cells inhibit the function of tumor-specific T cells through changes in immune checkpoint mechanism. In other words, the inhibition of tumor-specific T cells is avoided by activating these inhibitory immune checkpoints in tumor cells (Chen and Han 2015). Recently, anti-tumor effect can be obtained by suppressing its function by using monoclonal antibody against CTLA-4, PD-1 or PD-L1, thereby, enhancing the tumor-specific T cell activity and effect.

The aforementioned FDA-approved agents or other agents blocking immune checkpoints do not directly target cancer cells, but instead target molecules that are involved in the regulation of T cells involved in immune activity (Le et al. 2015; Phan et al. 2003). The main goal is to treat cancer by blocking the pathway of interfering with the T cells rather than directly attacking cancer cells.

**Fig. 4:**
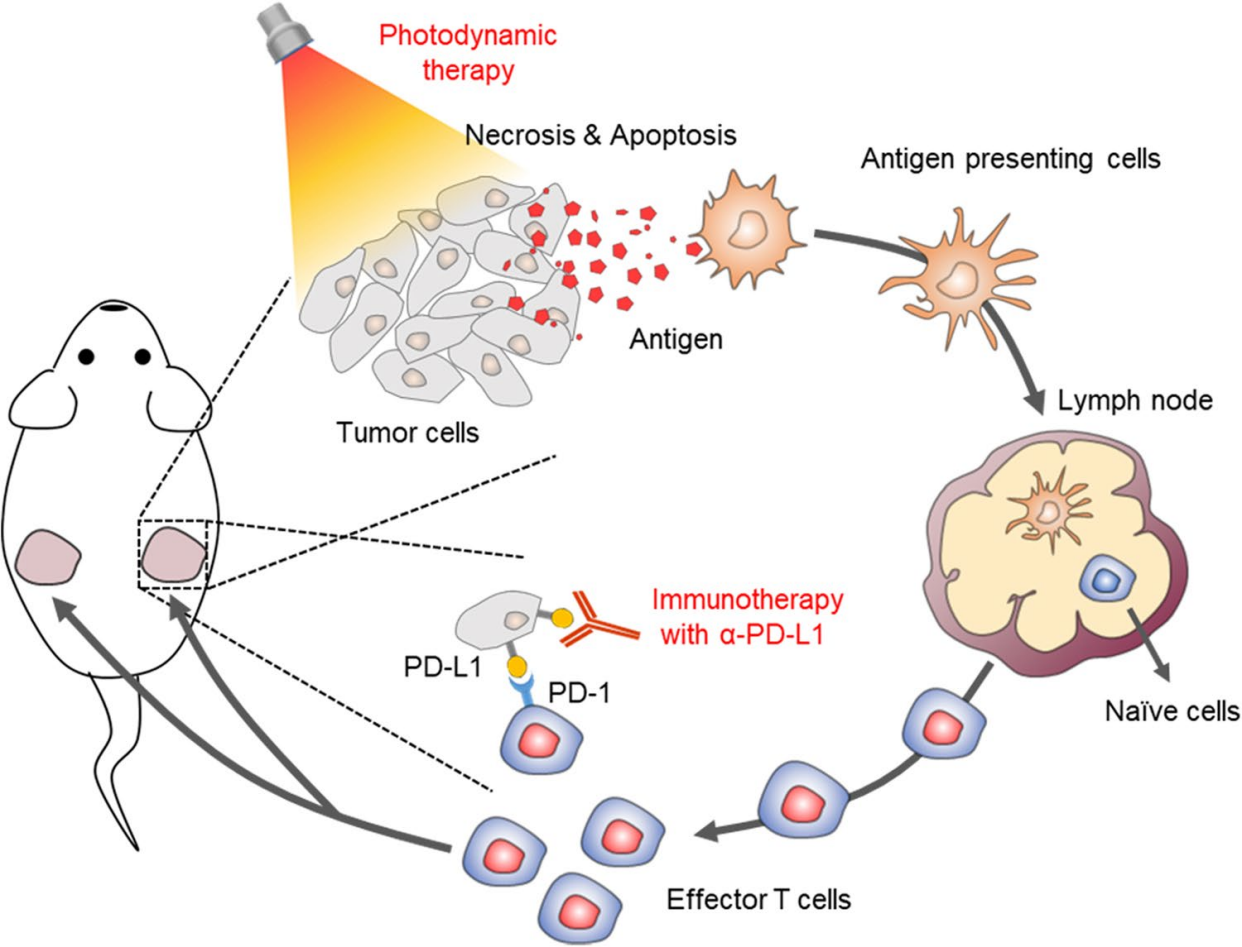
Chemotherapy and PDT potentiate PD-L1 blockade to induce systemic antitumor immunity. (Modified with permission from *Nat Commun* Copyright 2016)

Recently, Ralph and Wenbin reported the use of immunogenic nanoparticles to augment the antitumor efficacy of PD-L1 antibody-mediated cancer immunotherapy (He et al. 2016). They designed the nanoscale coordination polymer (NCP) core–shell nanoparticles carry oxaliplatin in the core for immunotherapy and the photosensitizer-lipid conjugate in the shell (NCP@pyrolipid) for effective chemotherapy and PDT. This NCP@pyrolipid exhibited the synergy effects between oxaliplatin and pyrolipid-induced PDT which kills tumor cells and induces an immune activity, resulting in calreticulin exposure on the cell surface, antitumor vaccination, and an abscopal effect. As they mentioned, when combined with anti-PD-L1 therapy, NCP@pyrolipid mediates regression of both light-irradiated primary tumors and non-irradiated distant tumors by inducing a strong tumor-specific immune response (Fig. 4).

As such, trends are now being investigated as a combination therapy to improve the anti-tumor effect by combining field of immune check points and other fields, such as PDT, PTT or radiation therapy.

## Conclusions

PDT-mediated immunotherapy is one of the promising therapeutic modalities that have been used to treat tumors. After PDT treatment, tumors are able to undergo three types of cell death pathways and make itself venerable to evoke antitumor immune response followed by tumor destruction. Although PDT-induced immune response is difficult to define because of the complexity of tumor microenvironments and involvement of many cytokines and immune cells, it is obvious that PDT provides effective immune induction and brings better outcomes by maximizing local inflammatory reaction and activation of immune cells to destruct tumor tissues. Moreover, there are possibilities that combination of PDT and chemo/radiotherapy will lead to improvement of antitumor immune response and therapeutic efficacy enhancement.
